# Correction to: Relationship between ventilator-associated pneumonia and mortality in COVID-19 patients: a planned ancillary analysis of the coVAPid cohort

**DOI:** 10.1186/s13054-021-03713-3

**Published:** 2021-08-09

**Authors:** Saad Nseir, Ignacio Martin-Loeches, Pedro Povoa, Matthieu Metzelard, Damien Du Cheyron, Fabien Lambiotte, Fabienne Tamion, Marie Labruyere, Demosthenes Makris, Claire Boulle Geronimi, Marc Pinetonde Chambrun, Martine Nyunga, Olivier Pouly, Bruno Mégarbane, Anastasia Saade, Gemma Gomà, Eleni Magira, Jean-François Llitjos, Antoni Torres, Iliana Ioannidou, Alexandre Pierre, Luis Coelho, Jean Reignier, Denis Garot, Louis Kreitmann, Jean-Luc Baudel, Guillaume Voiriot, Damien Contou, Alexandra Beurton, Pierre Asfar, Alexandre Boyer, Arnaud W. Thille, Armand Mekontso-Dessap, Vassiliki Tsolaki, Christophe Vinsonneau, Pierre-Edouard Floch, Loïc Le Guennec, Adrian Ceccato, Antonio Artigas, Mathilde Bouchereau, Julien Labreuche, Alain Duhamel, Anahita Rouzé, the coVAPid study group, Raphaël Favory, Sébastien Préau, Mercé Jourdain, Julien Poissy, Piehr Saint Leger, Thierry Van der Linden, Anne Veinstein, Elie Azoulay, Frédéric Pene, Maelle Martin, Keyvan Razazi, Gaëtan Plantefeve, Muriel Fartoukh, Didier Thevenin, Bertrand Guidet, Nicolas Weiss, Achille Kouatchet, Charlotte Salmon, Guillaume Brunin, Safaa Nemlaghi, David Meguerditchian, Laurent Argaud, Sebastian Voicu, Charles-Edouard Luyt, Benjamin Kowalski, Edgar Moglia, Luis Morales, Antonia Koutsoukou, Spyros D. Mentzelopoulos, David Nora, Sean Boyd, Julien Maizel, Pierre Cuchet, Quentin Delforge, Jean-Pierre Quenot, Déborah Boyer, Catia Cilloniz

**Affiliations:** 1 Médecine Intensive-Réanimation, CHU de Lille, F-59000 Lille, France; 2 Inserm U1285, CNRS, UMR 8576-UGSF-Unité de Glycobiologie Structurale et Fonctionnelle, Univ. Lille, Lille, France; 3 Department of Intensive Care Medicine, Multidisciplinary Intensive Care Research Organization (MICRO), St. James’s Hospital, St. James Street, Dublin 8, Dublin, Eire Ireland; 4 Hospital Clinic, IDIBAPS, Universided de Barcelona, CIBERes, Barcelona, Spain; 5 Polyvalent Intensive Care Unit, São Francisco Xavier Hospital, Centro Hospitalar de Lisboa Ocidental, and NOVA Medical School, CHRC, New University of Lisbon, Lisbon, Portugal; 6 Center for Clinical Epidemiology and Research Unit of Clinical Epidemiology, OUH Odense University Hospital, Odense, Denmark; 7 Medical ICU, Amiens University Hospital, Amiens, France; 8 Department of Medical Intensive Care, Caen University Hospital, 14000 Caen, France; 9 Service de Réanimation Polyvalente, Centre Hospitalier de Valenciennes, Valenciennes, France; 10 Medical Intensive Care Unit, Rouen University Hospital, Normandie Université, UNIROUEN, Inserm U1096, FHU-REMOD-VHF, 76000 Rouen, France; 11 Department of Intensive Care, François Mitterrand University Hospital, Dijon, France; 12 Intensive Care Unit, University Hospital of Larissa, University of Thessaly, 41110 Biopolis Larissa, Greece; 13 Service de Réanimation Et de Soins Intensifs, Centre Hospitalier de Douai, Route de Cambrai, Douai, France; 14 Service de Médecine Intensive Réanimation, Institut de Cardiologie, Hôpital Pitié-Salpêtrière, Assistance Publique-Hôpitaux de Paris (APHP), Sorbonne Université, 47-83, Boulevard de L’Hôpital, 75651 Paris Cedex 13, France; 15 ICU, Roubaix Hospital, Roubaix, France; 16 Médecine Intensive Réanimation, Hôpital Saint Philibert GHICL, Université Catholique, Lille, France; 17 Réanimation Médicale Et Toxicologique, Hôpital Lariboisière, Université de Paris, INSERM UMRS-1144, Paris, France; 18 Service de Médecine Intensive Et Réanimation, Hôpital Saint-Louis, 1 Avenue Claude Vellefaux, 75010 Paris, France; 19 Critical Care Department, Hospital Universitari Parc Taulí, Sabadell, Spain; 20 1St Department of Intensive Care Medicine, National and Kapodistrian University of Athens Medical School, Evaggelismos Hospital, Athens, Greece; 21 Medical Intensive Care Unit, Cochin Hospital, AP-HP. Centre, Université de Paris, Paris, France; 22 Department of Pulmonology, Hospital Clinic Barcelona, University of Barcelona, IDIBAPS, CIBERES, ICREA, Barcelona, Spain; 23 1St Department of Pulmonary Medicine and Intensive Care Unit, National and Kapodistrian University of Athens, “Sotiria” Chest Hospital, Athens, Greece; 24 Réanimation Polyvalente, CH Lens, Lens, France; 25 Service de Médecine Intensive Réanimation, CHU de Nantes, Nantes, France; 26 Service de Médecine Intensive Réanimation, CHU de Tours, Hôpital Bretonneau, 2 Bd Tonnellé, 37000 Tours, France; 27 Service de Médecine Intensive - Réanimation, Hospices Civils de Lyon, Hôpital Edouard Herriot, 5, place d’Arsonval, 69437 Lyon Cedex 03, France; 28 Service de Médecine Intensive Réanimation, AP-HP, Hôpital Saint-Antoine, Assistance Publique-Hôpitaux de Paris, 184 rue du Faubourg Saint-Antoine, 75571 Paris Cedex 12, France; 29 Sorbonne Université, Assistance Publique-Hôpitaux de Paris, Service de Médecine Intensive Réanimation, Hôpital Tenon, Paris, France; 30 Réanimation Polyvalente, CH Victor Dupouy, Argenteuil, France; 31 Service de Pneumologie, Médecine Intensive - Réanimation (Département “R3S”), AP-HP, Sorbonne Université, Groupe Hospitalier Universitaire Pitié-Salpêtrière Charles Foix, INSERM, UMRS1158 Neurophysiologie Respiratoire Expérimentale Et Clinique, Paris, France; 32 Département de Médecine Intensive-Réanimation, CHU D’Angers, Université D’Angers, 4 rue Larrey, 49933 Angers Cedex 9, France; 33 Intensive Care Unit, Pellegrin-Tripode Hospital, University Hospital of Bordeaux, Bordeaux, France; 34 CHU de Poitiers, Médecine Intensive Réanimation, CIC 1402 ALIVE, Université de Poitiers, Poitiers, France; 35 APHP, CHU Henri Mondor, Service de Médecine Intensive RéanimationUniversité Paris Est-Créteil, Faculté de Santé, Groupe de Recherche Clinique CARMASINSERM U955, Institut Mondor de Recherche Biomédicale, 94010 Créteil, France; 36 Service de Médecine Intensive Réanimation, Centre Hospitalier de Béthune, Réseau de Recherche Boréal, 62408 Béthune, France; 37 Service de Réanimation, Hôpital Duchenne, Rue Monod, 62200 Boulogne-sur-Mer, France; 38 Sorbonne Université, AP-HP, Hôpital de La Pitié-Salpêtrière, Département de Neurologie, Unité de Médecine Intensive Réanimation Neurologique, Paris, France; 39 Intensive Care Unit, Hospital Universitari Sagrat Cor, and Ciber de Enfermedades Respiratorias (Ciberes, CB06/06/0028)-Institut D’Investigacions Biomèdiques August Pi I Sunyer (IDIBAPS), Barcelona, Spain; 40 Critical Care Center, Corporacion Sanitaria Universitaria Parc Tauli, CIBER Enfermedades Respiratorias, Autonomous University of Barcelona, Parc Tauli 1, 08028 Sabadell, Spain; 41 Univ. Lille, CHU Lille, ULR 2694-METRICS: Évaluation Des Technologies de Santé Et Des Pratiques Médicales, 59000 Lille, France

## Correction to: Crit Care (2021) 25:177. https://doi.org/10.1186/s13054-021-03588-4

Following publication of the original article [1], the authors identified an error in the Abstract and Results section. The correct numbers and text are given hereafter.

**Abstract**

VAP was associated with significantly higher risk for 28-day mortality in SARS-CoV-2 group (adjusted HR 1.65 (95% CI 1.11–2.46), p = 0.013), but not in influenza (1.74 (0.99–3.06), p = 0.052), or no viral infection groups (1.13 (0.68–1.86), p = 0.63).

**Results**

Primary and secondary outcomes: VAP was associated with higher risk for 28-day mortality in in SARS-CoV-2 group, but not in the two other groups (Fig. 2A).

All the changes that were requested are implemented in this correction and the original article [1] has been corrected.

## References

[1] Nseir S, Martin-Loeches I, Povoa P (2021). Relationship between ventilator-associated pneumonia and mortality in COVID-19 patients: a planned ancillary analysis of the coVAPid cohort. Crit Care.

